# Genomic Insights Into Multidrug‐Resistant Foodborne *Serratia liquefaciens* Strains Carrying *mcr-9* and Comparative Genomic Analysis of Novel Biosynthetic Gene Clusters

**DOI:** 10.1155/ijfo/5035164

**Published:** 2026-09-15

**Authors:** Christian Xedzro, Toshi Shimamoto, Ashraf M. Ahmed, Liansheng Yu, Yo Sugawara, Motoyuki Sugai, Tadashi Shimamoto

**Affiliations:** ^1^ Laboratory of Food Microbiology and Hygiene, Graduate School of Integrated Sciences for Life, Hiroshima University, Higashihiroshima, Japan, hiroshima-u.ac.jp; ^2^ Department of Bacteriology, Mycology and Immunology, Faculty of Veterinary Medicine, Kafrelsheikh University, Kafr El-Sheikh, Egypt, kfs.edu.eg; ^3^ Antimicrobial Resistance Research Center, National Institute of Infectious Disease, Japan Institute for Health Security, Higashimurayama, Tokyo, Japan, nih.go.jp

**Keywords:** biosynthetic gene cluster, *mcr-9*, meat, multidrug resistance, *qseBC*

## Abstract

*Serratia liquefaciens* is an opportunistic nosocomial pathogen with a wide range of antibiotic resistance patterns. This study reports the characterization of the first *mcr-9*–positive *S. liquefaciens* strains, 35E‐19E1 and CST‐066, isolated from meat products in Japan. The strains were screened for the presence of *β*‐lactamases, plasmid‐mediated mobile colistin resistance (*mcr*) genes, and carbapenemase‐encoding genes using PCR. Antimicrobial susceptibility was tested using the broth microdilution method. The strains exhibited multidrug resistance (MDR) phenotypes to third‐generation cephalosporins, cephamycin, fosfomycin, and other clinically important antimicrobials. Genomic DNA sequencing showed that the genome sizes of CST‐066 and 35E‐19E1 are 5,529,704 and 5,261,506 bps, respectively. *mcr-9* was identified on a chromosome within a genetic environment that included the two‐component system *qseBC*, which plays a key role in the signaling network that triggers colistin resistance in Enterobacterales. Downstream genome analysis revealed a 1695‐bp eptB‐like kdo2‐lipid phosphoethanolamine transferase, which is involved in intrinsic polymyxin resistance mechanisms in *Serratia* spp. The strain 35E‐19E1 carries five CRISPR‐Cas enzymes that are essential for adaptive immunity in bacteria, allowing defense against invading elements. Functional analysis using subsystem technology revealed that both strains possess subsystem features responsible for invasion and adhesion within the host biomes. Genome mining using antiSMASH and BAGL4 revealed various biosynthetic gene clusters, responsible for secondary metabolite synthesis. Notably, we identified novel gene clusters, mainly nonribosomal peptide synthetases, in both the strains, indicating their potential to produce bioactive compounds. Although the presence of *mcr-9* in *Serratia* may not be of clinical significance because of natural resistance of the strain to polymyxins, we shed light on the genomic characteristics of this MDR pathogen and the potential spread of *mcr-9* among other bacterial species. The emergence of *mcr-9* in drug‐resistant *S. liquefaciens* provides significant insights, underscoring the need for increased surveillance of this pathogen.

## 1. Introduction

*Serratia* species are motile, anaerobic, facultatively aerobic, and nonsporulating Gram‐negative bacteria [1]. Taxonomically, the genus *Serratia* currently belongs to the Yersiniaceae family [2, 3] and comprises at least 14 species and two subspecies [4, 5]. Among the species, *Serratia marcescens* is well known to cause human infections, followed by *S. liquefaciens* [5, 6]. Although members of this genus were initially considered nonpathogenic, sporadic reports in hospitals and medical literature reveal that they are opportunistic and nosocomial pathogens capable of causing clinical diseases, such as urinary tract infections and pneumonia [4, 7, 8]. *Serratia* species are ubiquitous in nature and are commonly isolated from water, soil, plants, food, and moist environments [9, 10]. They possess several virulence factors and present acquired and inherent antimicrobial resistance mechanisms that reduce their susceptibility to aminoglycosides, *β*‐lactams, macrolides, cephalosporins, cationic antimicrobial peptides, and fluoroquinolone antibiotics [11, 12]. As such, the clinical management of *Serratia* has become a challenge, and severe treatment failures are expected to become a threat in clinical settings.

*S. liquefaciens* is pathogenic to humans and insects [4] and can attach to meat surfaces, equipment, and processing environments. These bacteria secrete extracellular polymers that facilitate the formation of dense biofilms, thereby providing protection against mechanical stress and antimicrobial agents [13]. The structure of the biofilm serves as a protective barrier against antimicrobial agents, enabling the resident bacteria to withstand removal forces. They also aid in the defense against host‐specific and nonspecific defense mechanisms, as well as harmful agents from the external environment [14]. Unlike *S. marcescens*, *S. liquefaciens* is associated with sepsis and bloodstream infection [6]. An outbreak of hospital‐acquired urinary tract infections caused by *S. liquefaciens* has been documented [15], highlighting the clinical importance of this pathogen. Antibiotics are used to treat bacterial infections; however, *S. liquefaciens* is naturally resistant to oxacillin, benzylpenicillin, cefazolin, cefaclor, cefuroxime, lincosamides, macrolides, streptogramins, fusidic acid, glycopeptides, and rifampicin [6], which complicates the effectiveness of clinical anti‐infective treatments.

The increasing occurrence of multidrug‐resistant (MDR) bacteria, driven by the acquisition of exogenous genes and bacterial exposure to antibiotics, which select for numerous resistance phenotypes, has been a global burden for decades [12]. The situation worsens when bacterial species acquire resistance to third‐generation cephalosporins and last‐resort antibiotics, such as carbapenems and colistin. Colistin is a last‐resort antibacterial drug used to treat patients with severe MDR bacterial infections [16, 17]. The emergence of plasmid‐mediated mobile colistin resistance (*mcr*) genes has garnered significant attention and is a topic of worldwide discussion. The *mcr* gene encodes phosphoethanolamine transferase, which alters the lipid A component of lipopolysaccharide and diminishes its negative charge affinity for colistin [18]. Since the first identification of *mcr-1* in China [19], numerous studies have described the emergence of *mcr-1* to *mcr-10* in diverse Enterobacteriaceae from different geographic locations [18, 20–24]. The *mcr-9* gene was first identified in *Salmonella enterica* serotype Typhimurium in the United States in 2019 [21]. Since then, this gene has disseminated worldwide with reported uncertainties as to what phenotype it may carry [17, 25]. In a recent surveillance study, we reported the emergence of *mcr-9* in *Raoultella ornithinolytica* isolated from vegetable produce [17], suggesting that this gene may have been present in emerging bacterial pathogens.

To the best of our knowledge, only one study has identified *mcr-9* in *Serratia* [26]. Although the reports on *mcr-9*–producing bacteria are increasing, studies on the genome and genetic diversity of *S. liquefaciens* remain largely unexplored. Researchers have reported clinical experiences with urinary tract infections caused by *Serratia*; however, genomic information on this strain is limited. Currently, the World Health Organization has included *Serratia* in the priority pathogen list and has designated it as a priority research area for developing alternative antimicrobial agents [27]. Microorganisms produce pigments that can serve as potential sources of novel antimicrobial drugs. They produce bioactive compounds with significant pharmaceutical potential because of their metabolic capabilities and ecological relationships. For example, the biosynthetic gene clusters (BGCs), serrawettin W1 and W2, identified in *S. liquefaciens* exhibit antimicrobial activity [28]. Aryl polyenes, a class of bacterial pigments, possess antioxidant properties and have efficacy against chronic and degenerative diseases [29]. Given the limited research on this topic within the genus *Serratia*, we performed a comparative genomic analysis to explore the presence of BGCs in the genomes of *S. liquefaciens*. This exploration is crucial for elucidating unknown or novel gene clusters and improving infection control interventions. The objective of this study is to comprehensively analyze the genomic profiles of two drug‐resistant foodborne *S. liquefaciens* strains isolated from meat products using whole‐genome sequencing.

## 2. Materials and Methods

### 2.1. Bacterial Strains and Identification

The two bacterial isolates, CST‐066 and 35E‐19E1, were laboratory stocks that were previously isolated from retail meat products in Hiroshima, Japan. The strain CST‐066 was isolated from a chicken meat sample collected in 2021. For the isolation, 25 g of the meat sample was homogenized with 225 mL of buffered peptone water (Nissui Pharmaceutical Co. Ltd., Tokyo, Japan). Then, 100 *μ*L of the suspension was plated on MacConkey (Eiken Chemical Co. Ltd., Tochigi, Japan) supplemented with 2 *μ*g/mL colistin. Strain 35E‐19E1 was recovered from beef imported to Japan in 2009. For bacterial isolation, 500 *μ*L of the meat juice was enriched in Luria–Bertani (LB) broth (Nacalai Tesque Inc., Kyoto, Japan) for 15 h and plated on MacConkey agar without antibiotic supplementation. The two strains were identified by amplifying and sequencing the 16S rRNA fragments using the universal primer sets 27F and 1492R. They were screened for the presence of acquired carbapenemases (*bla*_KPC_, *bla*_NDM_, *bla*_OXA-48_, *bla*_VIM_, and *bla*_IMP_) [30], *β*‐lactamases (*bla*_OXA_, *bla*_SHV_, *bla*_CTX-M_, *bla*_TEM_, and *bla*_AmpC_) [31], and *mcr* genes (*mcr-1* to *mcr-10*) [32–34].

### 2.2. Antimicrobial Susceptibility Testing

The isolates were tested for susceptibility or resistance to 15 antibiotics, namely ampicillin, cefotaxime, ceftriaxone, ceftazidime, cefoxitin, meropenem, kanamycin, gentamicin, chloramphenicol, ciprofloxacin, norfloxacin, trimethoprim, tetracycline, fosfomycin, and colistin (CST). The broth microdilution method recommended by the Clinical and Laboratory Standards Institute was used to determine the minimum inhibitory concentration (MIC) of each antibiotic. EUCAST breakpoint was employed for colistin MIC interpretation. Briefly, twofold serial dilutions of each antimicrobial agent were prepared in cation‐adjusted Mueller–Hinton broth (Becton, Dickson and Company, Sparks, Maryland), which was previously dispensed into a 96‐well flat‐bottom plate. Each well was inoculated with 5 *μ*L of bacterial suspension previously adjusted to a 0.5 McFarland standard. The plates were incubated at 37°C for 18 h with *Escherichia coli* ATCC 25922 serving as the quality control strain.

### 2.3. Biofilm Assay

Crystal violet staining [35] was used to determine the biofilm‐forming ability of the strains. Overnight, *Serratia* cultures were diluted (1:100) in LB medium, and 200 *μ*L of the suspension was pipetted into a V‐bottom 96‐well microtiter plate (Micro test plate 96‐well; Nerbe Plus, Germany). After incubation at 37°C for 24 h, planktonic cells were carefully removed, and the wells were washed three times with distilled water. The adhering biofilms in the wells were stained with crystal violet (0.1%, w/v) for 15 min and washed thrice with distilled water. Stained biofilms were solubilized in 95% ethanol (v/v). The relative biofilms were quantified by measuring the optical density at 570 nm using a microplate reader (Multiskan Sky; Thermo Scientific, Finland). We included a cefotaxime‐resistant *S. fonticola* for intragenus comparison with *S. liquefaciens* to distinguish whether biofilm phenotypes are shared traits or species‐specific within the *Serratia* genus.

### 2.4. Whole‐Genome Sequencing and Bioinformatic Analysis

Given its clinical importance and considering that *mcr-9* in *S. liquefaciens* has not been reported, total genomic DNA was prepared using the Qiagen Genomic‐tip 20/G kit (Qiagen, Inc.) according to the manufacturer′s guidelines. The whole genomes of the two strains were sequenced using the Illumina MiSeq Reagent kit v3 (2 × 300 bp) (Illumina, San Diego, California, United States) and Oxford Nanopore platforms (Oxford Nanopore Technologies, Oxford, United Kingdom). For Illumina MiSeq (short reads), a DNA sequencing library was constructed using an Enzymatic 5× WGS fragmentation mix and WGS ligase, whereas a Rapid Barcoding Kit (SQK‐RBK004) was used to prepare the library for Nanopore sequencing (long reads). Raw sequence data were processed for quality checks using FastQC software (https://www.bioinformatics.babraham.ac.uk/projects/fastqc/). A de novo hybrid assembly of the sequence data was performed using Unicycler v0.4.8. (https://github.com/rrwick/Unicycler). The quality of the assembled genomes was estimated using QUAST [36] and CheckM v1.1.3 [37], and only high‐quality contigs were used for subsequent analysis, although selected features from the original assembly were also reported where relevant. Functional annotation of the complete genome sequences of CST‐066 and 35E‐19E1 was performed using the DFAST [38] and RAST*tk* [39] bioinformatic tools. Antimicrobial resistance genes were identified using ResFinder v4.6.0 [40]. Pairwise average nucleotide identity (ANI) analysis was performed using JSpecies [41] to assess the taxonomic identity or genetic similarity with the type strain *S. liquefaciens* ATCC 27592 (Accession Number: CP006253.1). In silico DNA–DNA hybridization (dDDH) values for species delineation were calculated using a Genome‐to‐Genome Distance Calculator [42]. The > 95% ANI and > 70% dDDH values were used as cutoff to type the bacterial species. The CRISPR regions were identified using CRISPRCasFinder (https://crisprcas.i2bc.paris-saclay.fr/CrisprCasFinder/Index). The Easyfig tool was used to visualize and compare the genetic environment of *mcr-9* [43].

### 2.5. Phylogenetic Analysis

We selected complete bacterial genomes from the NCBI database and only assemblies belonging to the genus *Serratia* with estimated completeness ≥ 95% and contamination ≤ 5% were retrieved for analysis. A phylogenetic tree was constructed based on Genome‐BLAST Distance Phylogeny (GBDP) distances calculated from whole‐genome sequences using the Type Strain Genome Server (TYGS) (https://tygs.dsmz.de/background/show). Nineteen *Serratia* https://tygs.dsmz.de/background/show whole genomes, including CST‐066 and 35E‐19E1, were uploaded to the server for taxonomic analysis. For phylogenomic inferences, all pairwise comparisons of genomes were performed using GBDP. The trimming and distance formula *d5* algorithm was used to accurately infer intergenomic distances. TYGS compares the uploaded genomes with its database of type (strain) genomes and determines the most closely related type strain for the uploaded genomes. The core genome sequence phylogeny based on single‐nucleotide polymorphism (SNP) was constructed using CSIPhylogeny [44]. Interactive Tree of Life (iToL) v7.0 [45] and FigTree (http://tree.bio.ed.ac.uk/software/figtree/) were used to visualize the metadata in a midrooted phylogenetic tree.

### 2.6. Pan‐Genome Estimation

The core and accessory genes of *S. liquefaciens* and most common *Serratia* species were identified using Roary [46]. For comparative genome analysis, all sequences were reannotated using Prokka [47]. The output gff3 files were then downloaded and used as input data for pan‐genome analysis using Roary. The pan‐genome matrix was visualized using the Phandango server [48]. Additionally, the summary file generated using Roary software was used to assess the proportions of the pan‐genome.

### 2.7. BGC Prediction

The BGCs present in the genomes of CST‐066 and 35E‐19E1 were predicted using the antiSMASH 7.1.0 software (https://antismash.secondarymetabolites.org) and Bacteriocin Genome mining tool (BAGEL4) (http://bagel4.molgenrug.nl/). The chemical structures and IUPAC names of the metabolites identified using BAGEL4 were generated via ChemSpider (https://www.chemspider.com/).

### 2.8. Nucleotide Sequence Accession Number

The annotated genome sequences of CST‐066 and 35E‐19E1 were deposited in the DDBJ database under the BioSample Accession Numbers SAMD00853014 and SAMD00852976, respectively, under the BioProject Accession Number PRJDB19733.

## 3. Results

### 3.1. Identification of Bacteria and PCR Detection of Resistance Genes

The strains CST‐066 and 35E‐19E1 were identified as *S. liquefaciens* because they shared an ANI value > 98% with the type strain *S. liquefaciens* ATCC 27592. This value exceeded the ANI cutoff of > 95% to define the bacterial species. In the dDDH analysis, CST‐066 and 35E‐19E1 exhibited 89.6% and 93.6% similarity, respectively, with the type strain, which was higher than the dDDH cutoff of 70% for identifying bacterial species. These taxonomic metrics confirmed that the strains were *S. liquefaciens*. PCR amplification revealed that the isolates were negative for carbapenemases and *β*‐lactamase genes.

### 3.2. Antimicrobial Susceptibility Test and Biofilm Formation

MIC determinations showed that the two strains exhibited resistance to ceftriaxone, ceftazidime, cefoxitin, trimethoprim, tetracycline, fosfomycin, and colistin, but remained susceptible to the remaining antibiotics (Table 1). Although mentioning colistin resistance in this context may not have been essential because of the inherent resistance of the strains to this drug, we aim to highlight and update the distinctive characteristics of the genus from other Enterobacterales. Strain 35E‐19E1 was also resistant to chloramphenicol.

**Table 1:** Antimicrobial susceptibility profiles of *S. liquefaciens.*

Antimicrobial agent	Phenotype (MIC *μ*g/mL)		
	CST‐066	35E1‐19E1	*E. coli* ATCC 25922
Ampicillin	Sensitive (8)	Sensitive (8)	Sensitive (8)
Cefotaxime	Sensitive (1)	Sensitive (1)	Sensitive (0.25)
Ceftriaxone	Resistant (16)	Resistant (32)	Sensitive (0.5)
Ceftazidime	Resistant (> 128)	Resistant (128)	Sensitive (1)
Cefoxitin	Resistant (16)	Resistant (16)	Sensitive (4)
Meropenem	Sensitive (0.06)	Sensitive (1)	Sensitive (0.25)
Kanamycin	Sensitive (8)	Sensitive (8)	Sensitive (1)
Gentamicin	Sensitive (1)	Sensitive (1)	Sensitive (4)
Chloramphenicol	Sensitive (8)	Resistant (16)	Sensitive (8)
Ciprofloxacin	Sensitive (< 0.125)	Sensitive (< 0.125)	Sensitive (< 0.125)
Norfloxacin	Sensitive (< 0.125)	Sensitive (2)	Sensitive (0.063)
Trimethoprim	Resistant (128)	Resistant (128)	Sensitive (0.5)
Tetracycline	Resistant (8)	Resistant (8)	Sensitive (2)
Fosfomycin	Resistant (128)	Resistant (128)	Sensitive (16)
Colistin	Resistant (128)	Resistant (512)	Sensitive (0.5)

Crystal violet biofilm assay revealed significant variability in biofilm formation among the tested strains. Notably, strain CST‐066 exhibited a substantial increase in biofilm production compared with the other test strains (Kruskal–Wallis test, *p* < 0.001) (Figure S1). Dunn′s post hoc test with Bonferroni correction identified significant differences between CST‐066 and 35E‐19E1 (*p* < 0.001), CST‐066 and CH76‐1 (*p* < 0.001), and 35E‐19E1 and CH76‐1 (*p* = 0.016). The observed differences in biofilm mass among the strains may be linked to genome plasticity, which allows the regulation and restructuring of genetic content and can influence key biofilm‐related genes.

### 3.3. Genome Characterization of *mcr-9*–Harboring *S. liquefaciens*

The chromosome of strain CST‐066 is 5,529,704 bp long (55.3% GC content) and contains one contig with several RNAs (rRNA, tRNA, and tmRNA). This strain possesses 5340 coding sequences and carries *mcr-9*. In contrast, strain 35E‐19E1 contains a 5,261,506‐bp chromosome‐associated sequence, whereas the original genome assembly comprised 21 contigs containing 7790 predicted CDSs. The presence of the three resistance genes, *mcr-9*, *lsa*(A), and *clpL*, was confirmed using the ResFinder database, with each gene located on different contigs. We identified various CRISPR‐Cas genes in 35E‐19E1, which are involved in adaptive immunity in bacteria, and provide protection against invading genetic elements. The differences in genomic content between CST‐066 and 35E‐19E1 are presented in Table 2.

**Table 2:** Genomic features of *S. liquefaciens* analyzed in this study.

Category	CST‐066	35E‐19E1
Genome size bp	5,529,704	5,261,506
GC content (%)	55.3	55.4
Completeness	99.95	99.95
Contamination	1.38%	0.94%
Number of contigs	1	21*
Number of CDS	5340	7790*
Number of rRNA	22	34*
Number of tRNA	87	148*
Number of tmRNA	1	1
ANI (%)	98.14	98.75
Aligned nucleotides (%)	88.54	93.03
dDDH value (%)	89.6	93.6
RAST subsystems	388	382
AMR gene	*mcr-9*, *fosA*, *satA*	*mcr-9*, *lsa*(A)*, clpL*, *fosA*, *satA*
CRISPR‐Cas	0	5
Number of BGCs	64	55

*Note:* Bold value indicates the original total number of contigs in strain 35E‐19E1. For comparative genomic and downstream analyses, only high‐quality chromosome‐associated sequences were used. Features marked with asterisks are characteristics of the original genome assembly.

### 3.4. Genetic Context of *mcr-9*

**Figure 1:**
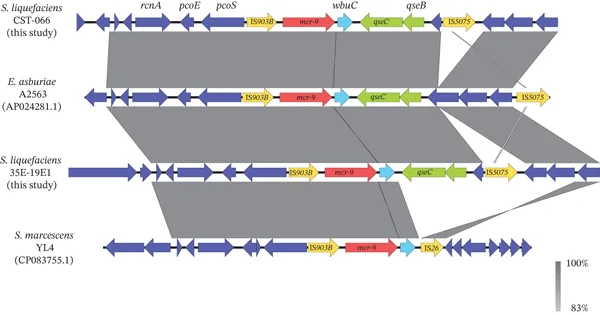
Comparison of the genetic environment of *mcr-9* across different bacterial species. The arrows indicate the positions and directions of the transcriptional orientations of the genes, and the shaded areas denote homologous regions (> 95% nucleotide identity).

We performed a comparative analysis of the genetic environments of two *mcr-9*–carrying isolates, *E. asburiae* A23563 (Accession No. AP024281.1) and *S. marcescens* YL4 (Accession No. CP083755.1), along with the isolates used in this study (Figure 1). A comparison of *mcr-9*–possessing regions among the isolates using the Easyfig tool revealed that they share homologous regions flanked by two mobile genetic elements (IS*903B* [IS5 family] and IS*5075* [IS110 family]) with 100% coverage and ~98% identity. In all four isolates, the upstream sequences of *mcr-9* were highly homologous, except for *S. marcescens* YL4, which contained an insertion of InsB. Downstream of *mcr-9* were unique open reading frames, including *wbuC* and *qseBC* genes. Although *wbuC* gene, a putative metalloenzyme of the cupin superfamily, remains functionally uncharacterized, a two‐component operon comprising the sensor kinase‐encoding gene *qseC* and response regulator‐encoding gene *qseB* has been shown to play a crucial role in the signaling network that triggers colistin resistance among Enterobacterales [49]. The structure of *rcnR*‐*rcnA*‐*pcoE*‐*pcoS*‐IS*903B*‐*mcr-9*‐*wbuC* is shared among the three isolates (Figure 1), providing additional evidence that these unique elements play essential roles in the processes of recombination, integration, and conjugation of *mcr-9* within bacterial genomes.

### 3.5. Annotations and RAST Subsystems

**Figure 2:**
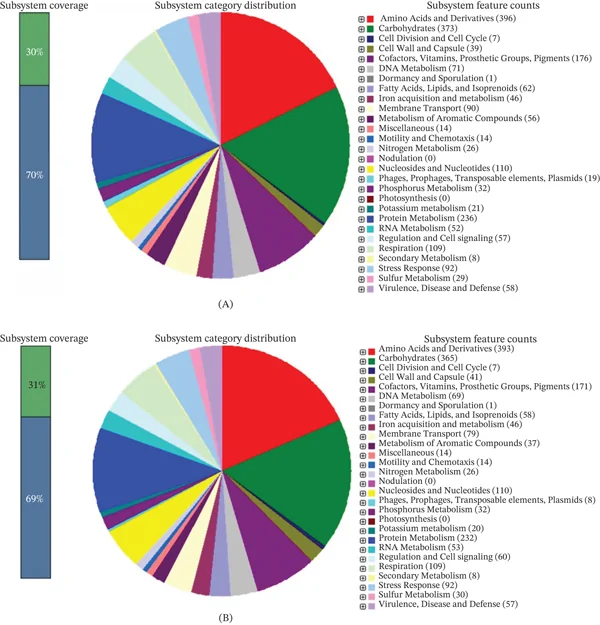
Pie chart displaying the RAST subsystem categories of *S. liquefaciens* (A) CST‐066 and (B) 35E‐19E1 genomes. The most prevalent systems in the category distribution are shown as bars on the left side of the pie chart, and the right column represents the number of subsystem features.

We used RAST to annotate the genes and coding sequences of CST‐066 and 35E‐19E1. In total, 388 and 382 subsystems were predicted for CST‐066 and 35E‐19E1, respectively, indicating that the metabolic parameters of the isolates varied. The predicted subsystems of these two strains are depicted in Figure 2. The top four subsystem features of the isolates include amino acid derivatives, carbohydrates, protein metabolism, and cofactors and vitamins. Other subsystem features include virulence, disease, and defense mechanisms. Downstream analysis of these subsystems revealed genes predicted to confer resistance to antibiotics and toxic compounds. Functional analysis of CST‐066 revealed genes associated with fosfomycin resistance (1), copper tolerance (9), copper homeostasis (16), cobalt–zinc–cadmium resistance (6), streptothricin resistance (1), adaptation to d‐cysteine (1), resistance to chromium compound (1), and *β*‐lactamase (1). Strain 35E‐19E1 possesses five MDR efflux pumps and genes associated with streptothricin resistance (1), adaptation to d‐cysteine (1), copper homeostasis (16), chromium resistance (1), fosfomycin resistance (1), copper resistance (10), bile hydrolysis (2), cobalt–zinc–cadmium resistance (5), and *β*‐lactam resistance (1). These subsystems represent genes that contribute to specific aspects of the organism physiology and metabolic activities.

### 3.6. Pan‐Genome and Phylogenomics

**Figure 3:**
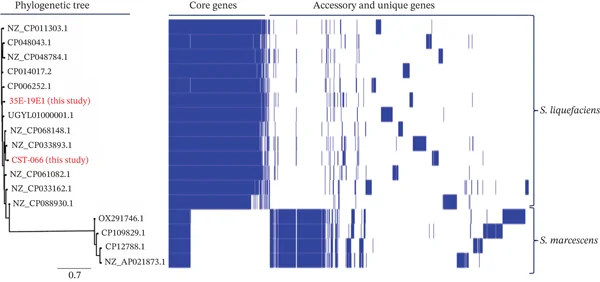
Pan‐genome matrix derived from the presence/absence of core and accessory genes of 17 *Serratia* spp., computed using Roary. Blue and white regions indicate gene presence and absence, respectively.

**Figure 4:**
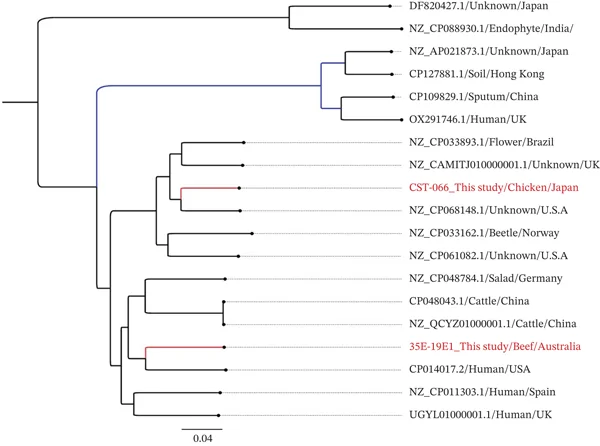
Maximum likelihood phylogeny of the core genomes of *Serratia* spp. constructed from the concatenated alignment of high‐quality SNPs. Strains analyzed in this study are shown by red highlights, whereas the blue branches represent *S. marcescens*.

We performed a pan‐genome analysis to establish the taxonomic relatedness between the isolates used in our study and those available in public databases. The genome data of 11 *S. liquefaciens* and four *S. marcescens* strains were obtained from a public database. Seventeen genomes were uploaded to the Roary server. Pan‐genome analysis showed that all 17 genomes shared 991 core genes with differences in gene presence or absence. The predicted pan‐genome size of *Serratia* spp. was 16,694 genes, of which 991 (6%) were present in all strains; 0 representing soft‐core genes; 6865 (41%) were shell genes; and 8838 (53%) represented cloud genes. Gene differences among the isolates included phage components, multidrug efflux pump proteins, and several hypothetical proteins. The gene presence or absence matrix plot (Figure 3) suggests that substantial differences exist between the isolates in this study and other *Serrati*a species. Phylogenomics based on the core‐genome alignment with SNP captured the genomic epidemiology of *S. liquefaciens* that formed different phylogenetic branches (Figure 4). Both the strains belong to distinct clades and have different host ranges in various parts of the world. The analysis showed that CST‐066 likely originated from plants or insects and that 35E‐19E1 was closely related to human isolates from the United States. A whole‐genome–based phylogeny was constructed, and the taxonomic identification of the query strains is shown in Figure S2.

### 3.7. Genome Mining and Novel BGC Identification

Genome mining using the antiSMASH and BAGEL tools identified the BGCs present in the genomes of both strains. The analysis revealed that CST‐066 and 35E‐19E1 harbored 64 and 55 BGCs, respectively (Table 2). We examined each gene cluster for homology with the reference strain *S. liquefaciens* ATCC 27592. All three strains shared six gene clusters associated with the biosynthesis of secondary metabolites, with conserved BGCs coding for beta‐lactone, aryl polyenes, photobactin, thiopeptide, Frederiksenbactin, and a nonribosomal peptide synthetase (NRPS)–like fragment (Table 3). The NRPS superfamily is common among the BGCs, making it a predominant class. The BGCs coding for aryl polyenes and Frederiksenbactin were identical in both strains, sharing 83% and 92% similarity, respectively, with those in the antiSMASH database. BAGEL4 analysis revealed that CST‐066–harbored carocin D, microcin C, and bottromycin bacteriocins, which were not detected in 35E‐19E1 or the reference strain (Figure S3). Although the structures of microcin and bottromycin were predicted, carocin D was not detected in the ChemSpider database. Notably, novel BGCs were identified in *S. liquefaciens* isolates. In CST‐066, three regions displayed less than 20% similarity to previously reported BGCs in the database, and the three regions showed no detectable similarity to NRPS. In 35E‐19E1, three unknown clusters with no similarity to known gene clusters were identified and two regions displayed < 20% similarity (Table 4). Both strains have two common novel gene clusters.

**Table 3:** Biosynthetic gene clusters (BGCs) of CST‐066, 35E‐19E1, and a reference strain.

Region/class	Most similar known cluster	Position of BGC		
		CST‐066	35E‐19E1	ATCC 27592
NRPS‐like fragment	NRPS‐like	451,640–494,621	465,280–508,261	421,156–464,137
Beta‐lactone	HGM‐like	727,887–753,530	700,212–725,859	658,054–683,701
Aryl polyenes, NI‐siderophore	Other	873,141–931,697	843,754–902,310	798,732–857,286
Photobactin	NRP/NRPS (Type 1)	949,629–994,172	920,655–965,198	872,382–916,924
Thiopeptide (O‐antigen)	Saccharide	1,893,272–1,919,730	1,882,781–1,909,238	1,703,213–1,729,670
Frederiksenbactin	NRPS	3,798,332–3,852,258	3,684,971–3,738,897	3,656,277–3,710,203

**Table 4:** Characterization of novel BGCs identified in CST‐066 and 35E‐19E1 with less than 20% similarity to those in the antiSMASH database.

Region	Region	Predicted substrate/polymer	Type	Similarity	Regulator (description)
CST‐066	1 (Cluster 1)	—	PKS + NRPS	—	CelR (cellobiose uptake repressor), ArgR (regulator of argine biosynthesis genes)
	2 (Cluster 2)	Phe‐Thr	NRPS	—	DasR (N‐acytylglucosamine dependent repressor)
	3		HMGL‐like	—	—
	5	Thr	NRP + NRPS	16%	ZuR (zinc‐responsive repressor), LexA (repressor of DNA damage response), AfsQ1 (two‐component system AfsQ1‐Q2 activator of antibiotic production)
	6	—	Saccharide	14%	—
	8 (Cluster 10)	Ser	NRPS + polyketide	14%	AfsQ1, ZuR, ArgR, NrdR (represses ribonucleotide reductase encoding genes)
35E‐19E1	1	—	Hserlactone, Autoind_synth	—	ZuR, NrdR
	2 (Cluster 2)	—	NRPS	—	CelR, ArgR
	3	—	Betalactone, HMG‐lilke	—	—
	5	Thr	NRP + NRPS	16%	ZuR, LexA, AfsQ1
	6	—	Saccharide	14%	—

## 4. Discussion

*S. liquefaciens* is an opportunistic and nosocomial pathogen that demonstrates intrinsic resistance to antibiotics, primarily owing to the presence of numerous efflux pumps [6]. Despite the clinical relevance of *Serratia* spp., epidemiological data on their antibiotic susceptibility profiles and genomic characterization are limited. Furthermore, detection of the plasmid‐mediated *mcr* gene, *mcr-9*, in *S. liquefaciens* has not been previously reported. Currently, one of the most pressing concerns is the increasing prevalence of plasmid‐borne *mcr* genes, which jeopardize the efficacy of colistin, one of the few last‐resort medications for treating severe MDR infections [17]. Additionally, the emergence of genes that confer antimicrobial resistance poses a significant challenge in the fight against drug‐resistant pathogens.

In this study, two antimicrobial‐resistant strains of *S. liquefaciens*, CST‐066 and 35E‐19E1, carrying the *mcr-9* gene, were isolated from chicken meat and characterized. Among the *mcr*‐like genes, *mcr-1* and *mcr-9* are widely distributed, with the *mcr-9* gene being identified in over 40 countries across six continents [50]. This indicates a rapidly growing dissemination of the *mcr-9* gene, which warrants further attention as it could pose a significant challenge to the effectiveness of clinical antibiotics if appropriate measures are not implemented. Phenotypic testing revealed that both isolates exhibited high resistance to colistin. Although *Serratia* is known to have natural resistance to polymyxins [51], the observed interstrain variation in MIC values remains unclear and warrants further investigation to identify the mechanisms underlying this phenotypic variation.

Cephalosporins are currently recommended as a treatment option for severe MDR infections [52, 53]. Therefore, highlighting the resistance of these isolates to third‐generation cephalosporins is important. Both isolates conferred clinical resistance to ceftriaxone and ceftazidime, yet demonstrated reduced susceptibility to cefotaxime, indicating a unique resistance phenotype of *S. liquefaciens*. Genome analysis using RAST identified the presence of *β*‐lactamase class C‐like enzymes and penicillin‐binding proteins from the superfamily, which may have contributed to cephalosporin resistance among the isolates. Various MDR efflux pumps may play dynamic roles, resulting in diverse patterns of cephalosporin resistance. Recently, cephalosporin‐resistant clinical *S. marcescens* has been reported in China [26], highlighting the potential for members of the *Serratia* genus to pose a significant challenge if future monitoring programs are compromised.

To assess whether biofilm formation in *Serratia* represents a shared genetic trait, we evaluated the biofilm‐forming capacities of the strains, including a cefotaxime‐resistant *S. fonticola* strain for intragenus comparison. Biofilm analysis using a crystal violet assay revealed appreciable biofilm‐forming ability across the strains, although the extent of biofilm formation varied among them. However, the inclusion of only a single *Serratia* comparator is insufficient to confirm whether biofilm formation is a conserved genetic characteristic across the genus. Nonetheless, we acknowledge this as a limitation of the study.

In the genomes of both isolates, a number of genes associated with resistance to streptothricin, fosfomycin, and *β*‐lactam antibiotics were identified. *satA*‐encoded streptothricin acetyltransferase A confers resistance to streptothricin by detoxifying it through acetylation of the beta amino group of the first beta‐lysyl moiety [54]. Similarly, *fosA* confers resistance to fosfomycin by catalyzing the conjugation of glutathione to carbon‐1 of the drug, which renders it ineffective as an antimicrobial agent [55]. Drug efflux systems are rapidly disseminated among Enterobacteriaceae members via conjugative plasmids and mobile genetic elements and play a crucial role in antimicrobial resistance [55]. Using subsystem technology, we identified the MATE family of MDR efflux pumps, specifically MdtK/YdhE/NorM, and the MdfA/Cmr major facilitator superfamily in the genome of *Serratia*. Additionally, other RND efflux pumps are recognized for their role as MDR transporters, and overexpression of this superfamily leads to increased resistance levels in bacteria. Genes associated with copper homeostasis were found in the genomes of both strains, with a gene cluster related to cobalt, zinc, and cadmium that included the respective *cusSR* two‐component systems, corroborating previously reported findings [56].

Analysis of the genetic environment surrounding *mcr-9* has uncovered genetic units that are also present in the plasmids of other *mcr-9*–carrying strains. *qseBC* genes are involved in triggering colistin resistance [17, 57]. In this study, we identified a similar functional genetic unit in which the *qseBC* operon was located downstream of *mcr-9*. The entire *mcr-9–qseBC* operon was flanked by two insertion sequences (ISs) belonging to the IS*5* and IS*110* families, suggesting the potential role of these mobile elements in the mobilization of the operon. Furthermore, the *mcr-9* gene may have been introduced into *S. liquefaciens* through these IS‐mediated transpositions. This phenomenon raises significant concerns because the *mcr-9* gene could potentially confer colistin resistance in other noncolistin‐resistant bacteria via horizontal gene transfer, particularly when these genes are located on the plasmid.

From a genomic perspective, *mcr-9*–associated mobile elements exhibit significant plasticity. The gene is predominantly located on IncHI2 plasmids, where it resides within MDR regions alongside other resistance factors [17]. These regions are commonly flanked by ISs, such as IS*903B*, IS*5075*, or IS*26*, which facilitate genetic mobility. These mobile elements, along with integrons and integrative conjugative elements, frequently play a significant role in mediating the acquisition and mobilization of *mcr-9*, thereby promoting horizontal gene transfer among bacterial populations [21, 57]. It is worth noting that the consistent proximity of *mcr-9* to IS*903*‐like genetic element suggests that its acquisition is likely dependent on an IS*903*‐mediated mechanism. Comparative genomic analyses indicate that while Enterobacteriaceae often harbor *mcr-9* on conserved plasmid backbones [17], the genetic contexts surrounding the gene show greater diversity, a pattern also observed in non‐Enterobacteriaceae isolates (*Serratia* in Figure 1). This suggests multiple independent recombination and mobilization events in the bacterial genome. Overall, the evolution of *mcr-9*–carrying elements appears to be driven by a combination of modular gene exchange, recombination, and horizontal transfer mechanisms, facilitating their persistence and dissemination across diverse bacterial hosts. Understanding these genetic mechanisms is essential for developing strategies to monitor and control the spread of resistance genes in bacterial populations.

The pan‐genome analysis revealed significant taxonomic relationships and diversity among various *Serratia* spp., as determined using Roary. Notably, ~94% of the genome is composed of accessory genes, mainly large chromosomal regions of difference. These regions are often associated with phage elements, integrases, or transposases. Although core genes encode most metabolic and cellular processes, accessory genes may play a vital role in the long‐term infection of genetically diverse hosts [58]. Notably, only 6% of the genes were identified as part of the core genome across all strains, and 94% of the genes were part of the accessory genes (not shown). The substantial proportion of accessory genes highlights the adaptability of *S. liquefaciens* to various niches and functions. The prevalence of accessory genes in *Serratia* spp. is high [59], underscoring their essential roles in the adaptability, survival, and evolutionary success of these nosocomial strains. SNP‐based phylogenetic analysis has allowed the segregation of strains isolated from various sources worldwide. The two *S. liquefaciens* strains were closely related to foreign isolates, although they formed distinct clusters, indicating diverse evolutionary pathways and transmission routes [60]. The position of the CST‐066 strain in the SNP phylogeny suggests that this nosocomial strain likely originated from a nonclinical environment and therefore indicates that nonclinical environmental strains of *S. liquefaciens* may evolve into opportunistic pathogens in nosocomial infections. Conversely, 35E‐19E1 fell within a branch of the species isolated from human specimens, suggesting that this pathogen may have originated from human or clinical environments. Although we did not investigate virulence, the nosocomial strains possessed adhesion and invasins, indicating potential for host colonization and pathogenicity. Whole‐genome phylogenetic analysis was conducted using GBDP, which incorporates reference and representative genomes as part of a comprehensive phylogenomic genome analysis. The analysis indicated that the strains were members of the *Serratia* genus, clustered together within the same clade, and were distinctly separated from the out‐group bacterial strains. Given its position in the phylogenetic hierarchy, the clade comprising *S. fonticola* LMG 7882 appeared to be more closely related to the common ancestor of all *Serratia* spp. than to the other clades (Figure S2) [56].

Genome mining of *S. liquefaciens* revealed that it shares six BGCs that may contribute to the production of secondary metabolites. This finding indicated that both strains have the potential to produce a similar range of bioactive compounds. AntiSMASH analyses identified the common BGCs: beta‐lactone, aryl polyenes, photobactin, thiopeptide, Frederiksenbactin, and NRPS‐like fragment. Notably, the NRPS superfamily appeared to be predominant among the genes, including novel gene clusters. Previously, serrawettin W1 and W2, which are part of the NRPS gene cluster in *S. liquefaciens*, were identified by Marques‐Pereira et al. [28]. This suggests that members of this genus produce other BGCs to thrive in habitats characterized by hydrophobic, hydrophilic, and axenic conditions. The compounds identified in this study exhibited a range of biological properties. For example, beta‐lactone and photobactin have antimicrobial or antifungal properties [61, 62]. Aryl polyenes, which are bacterial pigments, remain poorly understood. However, they have been shown to possess antioxidant properties and are beneficial in combating chronic and degenerative diseases caused by oxidative stress [29]. Remarkably, BAGEL4 analysis identified additional BGCs in CST‐066, specifically bacteriocins (carocin, microcin, and bottromycin) that were not detected in 35E‐19E1 or the reference strain, suggesting the potential of these nosocomial strains to produce bioactive compounds. We were unable to further evaluate the biological properties of these BGCs, which is a limitation of our study.

## 5. Conclusion

In summary, we identified *mcr-9* in two MDR *S. liquefaciens* strains of meat origin. The genetic context of IS*903B*‐*mcr-9*‐*wbuC*‐*qseC*‐*qseB*‐IS*5075* was present in both strains, suggesting the potential for the transferability of *mcr-9* across bacterial populations. Pan‐genomic and phylogenomic analyses revealed the taxonomic identities and origins of these nosocomial pathogens. Focusing on the evolutionary pathways of drug‐resistant *Serratia* is necessary to fully understand how MDR *Serratia* evolve and disseminate across diverse microbial populations. We also discovered novel BGCs in the genomes of the isolates that exhibited less than 20% or no similarity to those in the antiSMASH database. Therefore, future studies should explore the AMR and BGC profiles of this pathogen in a broader context to uncover its true characteristics.

## Author Contributions

Christian Xedzro, Toshi Shimamoto, and Tadashi Shimamoto conceptualized the idea, conducted formal analysis, and managed data curation. Christian Xedzro performed the experiments, analyzed the data, and wrote the original draft. Toshi Shimamoto managed the project administration and visualization and reviewed the original draft. Ashraf M. Ahmed managed data curation and formal analysis. Liansheng Yu, Yo Sugawara, and Motoyuki Sugai performed whole‐genome sequencing and analysis, reviewed, and commented on the manuscript. Tadashi Shimamoto supervised the project, managed the project administration and visualization, designed the method, reviewed, and edited/prepared the final draft.

## Funding

This study was supported by Ministry of Health, Labour and Welfare (10.13039/501100003478; JP24KA1005).

## Ethics Statement

The authors have nothing to report.

## Conflicts of Interest

The authors declare no conflicts of interest.

## Supporting information


**Supporting Information** Additional supporting information can be found online in the Supporting Information section. The supporting file attached shows the differences in biofilm formation and whole‐genome–based phylogeny of *Serratia* spp. as well as predicted structures of bacteriocins.

## Data Availability

All data supporting the findings of this study are available within the paper and its supporting information.
