# Supplementary material for: Genomic Insights Into Multidrug‐Resistant Foodborne Serratia liquefaciens Strains Carrying mcr-9 and Comparative Genomic Analysis of Novel Biosynthetic Gene Clusters
Source: Int J Food Sci. 2026 Sep 15;2026:5035164. doi: 10.1155/ijfo/5035164 (PMC13575642; doi:10.1155/ijfo/5035164)
Supplement: Supplementary file 1 [file IJFO-2026-5035164-s001.docx]

**Figure S1.** Box plots representing biofilm formation by *Serratia* spp. The mean data points are indicated by red asterisks. Statistical significance was determined using the Kruskal-Wallis test (*p* < 0.001). Dunn’s post-hoc test with Bonferroni correction identified significant differences between CST-066 and 35E-19E1 (*p* < 0.001), CST-066 and CH76-1 (*p* < 0.001), and 35E-19E1 and CH76-1 (*p*=0.016). The cefotaxime-resistant *S. fonticola* CH76-1 was included for intra-genus comparison to assess whether biofilm phenotypes are shared genetic traits within the *Serratia* genus.

**Figure S2.** Whole-genome-based phylogeny inferred from GBDP distances calculated from genome sequences. The branch lengths are scaled in terms of the GBDP distance formula *d5,* and the number of branches is the support value of >60% from 100 pseudo-bootstrap replications

**Figure S3.** Predicted structures of bacteriocins and their verified IUPAC names. Carocin-D was not identified in the ChemSpider database.
